# Carboplatin and vinblastine monthly in the optic pathway and hypothalamic gliomas: A retrospective analysis in a single institute

**DOI:** 10.1093/noajnl/vdaf020

**Published:** 2025-01-29

**Authors:** Ting-Bin Lin, Chao-Yang Kuo, Feng-Chi Chang, Shih-Chieh Lin, Yi-Wei Chen, Muh-Lii Liang, Yi-Yen Lee

**Affiliations:** Division of Pediatric Neurosurgery, Department of Neurosurgery, Neurological Institute, Taipei Veterans General Hospital, Taipei, Taiwan; Graduate Institute of Artificial Intelligence and Big Data in Healthcare, Smart Healthcare Interdisciplinary College, National Taipei University of Nursing and Health Sciences, Taipei, Taiwan; School of Medicine, National Yang Ming Chiao Tung University, Taipei, Taiwan; Department of Radiology, Taipei Veterans General Hospital, Taipei, Taiwan; Department of Pathology and Laboratory Medicine, Taipei Veterans General Hospital, Taipei, Taiwan; School of Medicine, National Yang Ming Chiao Tung University, Taipei, Taiwan; Department of Heavy Particles and Radiation Oncology, Taipei Veterans General Hospital, Taipei, Taiwan; School of Medicine, National Yang Ming Chiao Tung University, Taipei, Taiwan; Department of Medicine, Mackay Medical College, New Taipei City, Taiwan; Department of Neurosurgery, Mackay Memorial Hospital, Taipei, Taiwan; School of Medicine, National Yang Ming Chiao Tung University, Taipei, Taiwan; Division of Pediatric Neurosurgery, Department of Neurosurgery, Neurological Institute, Taipei Veterans General Hospital, Taipei, Taiwan

**Keywords:** chemotherapy, optic pathway and hypothalamic glioma (OPHG), pediatric brain tumor

## Abstract

**Background:**

Chemotherapy plays an important role in the treatment of optic pathway hypothalamic gliomas (OPHGs). Commonly used regimens include carboplatin and vincristine and monotherapy with vinblastine weekly. In this retrospective study, we used a monthly regimen of carboplatin and vinblastine to treat progressive/recurrent OPHGs and evaluated their effectiveness, visual preservation, and toxicity.

**Methods:**

The study involved patients with OPGH who were treated with carboplatin and vinblastine once per month. The response, disease progression, overall survival, vision changes, and toxicity were recorded according to their medical charts at our institute, and survival was analyzed.

**Results:**

A total of 25 patients were included, including 15 males (60%) and 10 females (40%). The response rate was 11/25 (44%), and the stabilization rate (complete response rate + partial response rate + minor response rate + and stable disease rate) was 21/25 (84%). The 3-year progression-free survival (PFS) rate was 54.6%, and the 5-year PFS rate was 46.8%. The 5-year overall survival rate was 100%. There were 6 patients who showed improved visual acuity (28.6%). Stable vision was found in 52.4% of patients. Only 2 patients experienced severe allergic reactions to carboplatin.

**Conclusions:**

The results showed that extending the dosing interval of carboplatin and vinblastine to every month can be seen as a similar response compared with previous regimens. The toxicity of this regimen is milder, and patients benefit from a lower frequency of hospital visits. The regimen can be considered as a choice of the first line of chemotherapy for OPHG patients.

**Key Points**

1. The monthly carboplatin and vinblastine regimen is effective for OPHGs.
2. Better PFS is observed in chemotherapy-naive patients.
3. This approach balances treatment efficacy with minimizing disruptions to daily life and toxicities.

**Importance of the Study**

Chemotherapy plays an important role in the treatment of optic pathway hypothalamic gliomas (OPHGs). We have explored a modified approach using a combination of carboplatin (175 mg/m^2^) and vinblastine (6 mg/m^2^) every month for treating progressive/recurrent OPHG. This adjusted approach strikes a balance between treatment effectiveness and minimizing the disruption of patients’ daily lives as part of ongoing efforts to optimize chemotherapy protocols.

Optic pathway hypothalamic gliomas (OPHGs) primarily affect children and constitute 3%–5% of pediatric intracranial tumors of the central nervous system.^1^ Histopathologically, the majority of these tumors are low-grade gliomas and predominantly pilocytic astrocytomas classified as World Health Organization grade I.^2^ There is an association between OPHG and neurofibromatosis type 1 (NF1), where OPHGs affect 15%–25% of patients with NF1.^3,4^ Clinical symptoms of OPHGs depend on the tumor’s location and its impact on surrounding structures. Because of the location, it is not easy to resect completely, which makes treatment more difficult.

Current treatment options for OPHG include observation, chemotherapy, radiotherapy, surgery, and targeted therapy. Neurosurgery in OPHGs is primarily limited to obtaining biopsy samples or addressing complications related to the tumor or its treatment, due to the challenging location of these tumors.^5^ Historically, radiotherapy was a cornerstone treatment for progressive OPHGs.^6^ However, radiotherapy has been progressively abandoned in the management of pediatric patients with optic pathway gliomas, due to its long-term side effects such as vasculopathy, endocrine deficits, and cognitive impairment, particularly in young children. Furthermore, recent long-term follow-up studies have indicated that radiation therapy is associated with a greater risk of death.^7,8^ Therefore, chemotherapy plays a crucial role in OPHG treatment, especially considering its potential to avoid these long-term effects.

Current clinical trials are investigating targeted therapy focusing on the mitogen-activated protein kinase pathway and the mammalian target of the rapamycin (mTOR) pathway.^9–13^ The most common genetic alterations in pLGGs are BRAF fusion (KIAA1549-BRAF) and BRAF V600 E mutation.^14^ A study analyzing BRAFV600E in 1320 nervous-system tumors found that rather than BRAF fusion, BRAF V600E seems to be more frequent in extra-cerebellar pilocytic astrocytoma than in cerebellar tumors, especially in the diencephalic region (33%).^15^

Patients with genetic alteration who were treated with targeting drugs showed a response in these unresectable tumors. Bouffet et al. demonstrated that targeted therapy with BRAF inhibitors significantly improved progression-free survival (PFS) compared to conventional chemotherapy in the upfront setting for pLGGs with BRAF alterations.^13^ This finding has positioned targeted therapies as a new standard of care for such patients.

Various chemotherapy regimens have been widely studied for treating pLGGs, with most achieving 5-year PFS rates of about 30%–50%.^16–22^ Commonly used regimens include carboplatin and vincristine; thioguanine, procarbazine, lomustine, and vincristine (TPCV); and vinblastine monotherapy. When selecting a chemotherapy regimen, it is crucial to consider both short-term and long-term toxicity. Carboplatin is known for hypersensitivity reactions, which pose a significant concern in regard to short-term toxicity.^16–18^ In previous studies, vincristine was found to be associated with a high risk of neurotoxicity.^23^ In contrast, vinblastine has been shown to have significantly lower neurotoxicity compared to vincristine.^24^ However, previous Phase 2 studies have identified hematologic side effects as the primary toxicity of weekly vinblastine.^19,20^

Both carboplatin and vinblastine have demonstrated single-agent activity in children with low-grade gliomas. A phase 1 study has analyzed carboplatin and vinblastine regimens for patients with pLGGs, and the regimen of carboplatin (400 mg/m²) on day 1 + vinblastine (4.0 mg/m²) weekly × 3 every 4 weeks was recommended for a Phase 2 trial.^25^ Additionally, a retrospective study explored the regimen of carboplatin (400 mg/m²) on day 1 + vinblastine (4.0 mg/m²) weekly × 3 every 4 weeks and suggested that this chemotherapy regimen might result in comparable efficacy to other carboplatin and vincristine regimens with fewer hypersensitivity reactions.^26^

We have explored a modified approach with regard to the impact of weekly hospital visits on patients’ quality of life, the higher hypersensitivity associated with weekly carboplatin, as well as the hematotoxicity caused by weekly vinblastine. To reduce neurotoxicity, we replaced vincristine with vinblastine in the traditional carboplatin and vincristine regimen. The proposed regimen involves a combination of carboplatin (175 mg/m^2^) and vinblastine (6 mg/m^2^) every month to treat progressive or recurrent OPHG. These dosages are based on a combination of weekly carboplatin with vincristine along with weekly vinblastine administration. However, we have extended the dosing frequency to a monthly schedule.

Theoretically, the toxicity of this chemotherapy should be lower than that of conventional monthly carboplatin, weekly vinblastine, and a weekly carboplatin and vincristine combination. This adjusted approach strikes a balance between treatment effectiveness and minimizing the disruption to patients’ daily lives, as well as toxicity effects such as hypersensitivity and hematologic side effects. This approach reflects ongoing efforts to optimize chemotherapy protocols. We retrospectively reviewed the response, disease progression, OS, vision changes, and toxicity among patients with progressive or recurrent OPHG.

## Methods

In this retrospective study, we selected patients who were diagnosed with OPHG and treated at our institution between September 2009 and September 2021. We included only patients who had both imaging and pathological confirmation of OPHG. We excluded patients who were ultimately diagnosed with tumors other than OPHG based on pathology reports, as well as patients with NF1.

Chemotherapy was indicated for all included patients due to radiologic evidence of progression or recurrence of tumors or worsening clinical symptoms. According to our hospital’s longstanding protocol, all patients are required to sign a consent form for chemotherapy after the physician provides an explanation prior to starting a new chemotherapy regimen. Among the patients who received chemotherapy, only those treated with the carboplatin/vinblastine regimen were included, while patients receiving other chemotherapy regimens were excluded. Data for this study were extracted from the patient’s medical records at our institution. The study was approved by the Institutional Review Board of Taipei Veterans General Hospital.

Demographic data were collected, including the age at diagnosis, age at treatment, sex, pathology, and tumor location (stage 1, 2, or 3 according to the Dodge classification),^27^ and the number of patients with endocrinopathy. Patients were all included in the study regardless of whether or not they had previously received other chemotherapy regimens (chemotherapy-naïve or non-naïve). The data collected on clinical outcomes of therapy included the date of starting chemotherapy, the response to chemotherapy, the date of tumor progression, and the date of death. Changes in visual acuity were also recorded. Adverse events during every cycle of chemotherapy were reviewed.

Responses were evaluated based on revised criteria of the response assessment in neuro-oncology.^28^ Data were also collected about the time to tumor progression, time to death, and time to censoring from the date of starting chemotherapy to calculate the PFS rate and OS rate. The date of tumor progression was defined by the appearance of radiological evidence of tumor progression (PD) or rapid clinical deterioration with or without radiological evidence. As part of our hospital’s routine practice, we regularly hold multidisciplinary meetings during patients’ hospital stays, where radiologists assess tumor status, which is documented as part of the patient’s medical records. Additionally, radiologists were asked to re-confirm the tumor assessments for this study.

Visual preservation was evaluated using visual acuity reports to examine visual status after chemotherapy (improvement, stabilization, or deterioration). Improvement was defined as an advancement of at least 0.2 log MAR units on the log MAR scale. Deterioration was considered as a decline by the same amount. Toxicity was reviewed, and adverse events were recorded based on the Common Terminology Criteria for Adverse Events version 5.0 (CTCAE, V5.0). The number of patients who discontinued the regimen due to severe adverse events was recorded. Adverse events above grade 3 in particular were recorded for better toxicity evaluation.

Means and standard deviations were used to express continuous variables, and frequencies and percentages were calculated for categorical variables. Statistical significance was defined using *P < *.05. Kaplan–Meier curves and a log-rank test were used to assess PFS and OS. Hazard ratios and 95% confidence intervals (Cis) were calculated using Cox proportional hazard regression analysis. Statistical analyses were conducted using SAS 9.4 (SAS Institute).

## Results

### Patient Characteristics

**Figure 1.**
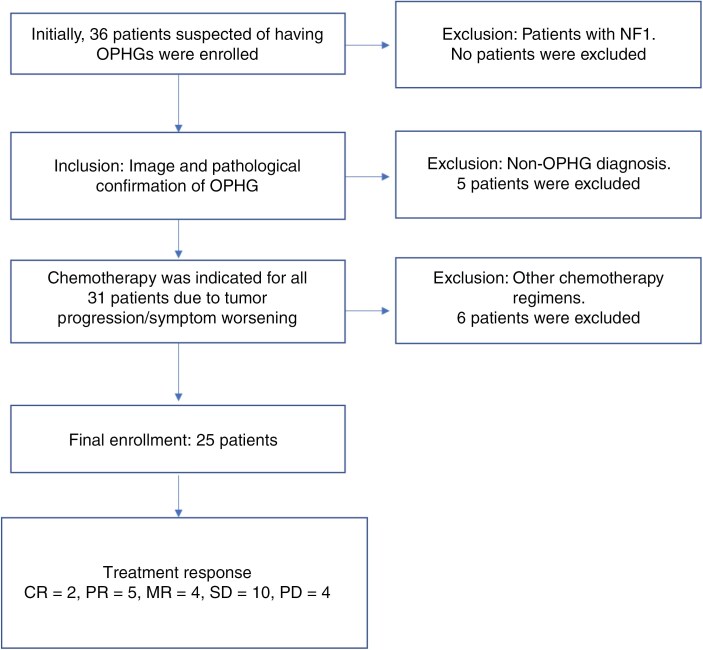
Flow diagram of patient selection and treatment response.

Initially, 36 patients who were suspected of having OPHG were included in the study. None of the 36 included patients had NF1. There were 5 patients who were diagnosed with tumors other than OPHG using imaging and pathology reports, which included one teratoma, one germinoma, one pineal region pilocytic astrocytoma, one nasal-cavity round-cell tumor, and one cardio-cervical junction chordoma. All of these patients were removed from this study. The remaining 31 patients had all undergone chemotherapy due to radiological evidence of tumor progression or worsening clinical symptoms. However, 6 patients who were treated with other regimens were excluded. Finally, 25 patients were included in the retrospective analysis. A flow diagram of the patient selection is shown in Figure 1.

There were 15 males (60%) and 10 females (40%). The age at the diagnosis ranged from 0.08 to 33.41 years. The median age was 4.88 years, and the mean was 6.78 years. There were 3 patients over 18 years old who were diagnosed at ages 23, 24, and 33, respectively. Two of them had pathological evidence of low-grade astrocytoma, and one had radiological evidence of OPGH.

There were 16 patients (64%) who had undergone craniotomy for tumor resection before the chemotherapy course. There were 2 cases that underwent near-total tumor resection (8%), 4 cases that underwent subtotal tumor resection (16%), and 8 cases that underwent partial tumor resection (32%). In 2 cases (8%), the degree of tumor resection was unknown because the surgeries were done at other hospitals without detailed medical records. In regard to histology, there were 12 pilocytic astrocytomas (48%) and 5 low-grade astrocytomas (20%).

There were 8 patients (32%) who had no tissue diagnosis. Molecular testing is not covered by Taiwan’s National Health Insurance and is not routinely performed. As a result, molecular data was unavailable for the majority of our patients. There were 8 patients (30.77%) who were non-naïve, and 17 patients (72%) who were chemotherapy-naïve. There were 7 patients (28%) who had endocrinopathy.

Due to the small number of patients, tumor locations were categorized using Dodge classification stages 1, 2, or 3 rather than the modified Dodge classification.^29,30^ There were 22 patients who were classified as stage 3 with hypothalamic involvement (88%). Only 2 patients were classified as stage 2 (8%), and 1 patient was classified as stage 1 (4%). Table 1 summarizes the overall characteristic distribution of the 25 patients.

**Table 1:** Characteristics of the Patients

Characteristic	No. of patients
Gender	
Male	15 (60%)
Female	10 (40%)
Age at diagnosis	
Mean	6.78 (years)
Median	4.88 (years)
Range	0.08~33.41 (years)
Age at starting carboplatin and vinblastine	
Mean	9.82 (years)
Median	8.86 (years)
Range	0.5~33.66 (years)
Pathology	
Pilocytic astrocytoma	12 (48%)
Grade 2 astrocytoma	5 (20%)
No	8 (32%)
Surgery	
yes	17 (68%)
Gross/near total	2 (8%)
Subtotal	4 (16%)
Partial	8 (32%)
Biopsy	1 (4%)
Unknown	2 (8%)
No	8 (32%)
Chemotherapy	
Chemotherapy-naive	17(68%)
Non-chemotherapy-naive	8(32%)
Location of Dodge classification	
Stage1	1(4%)
Stage2	2(8%)
Stage3	22(88%)
Leptomeningeal dissemination	
Yes	5(20%)
No	20(80%)
Endocrinopathy	
Yes	7(28%)
No	18(72%)

### Response to Treatment

**Figure 2.**
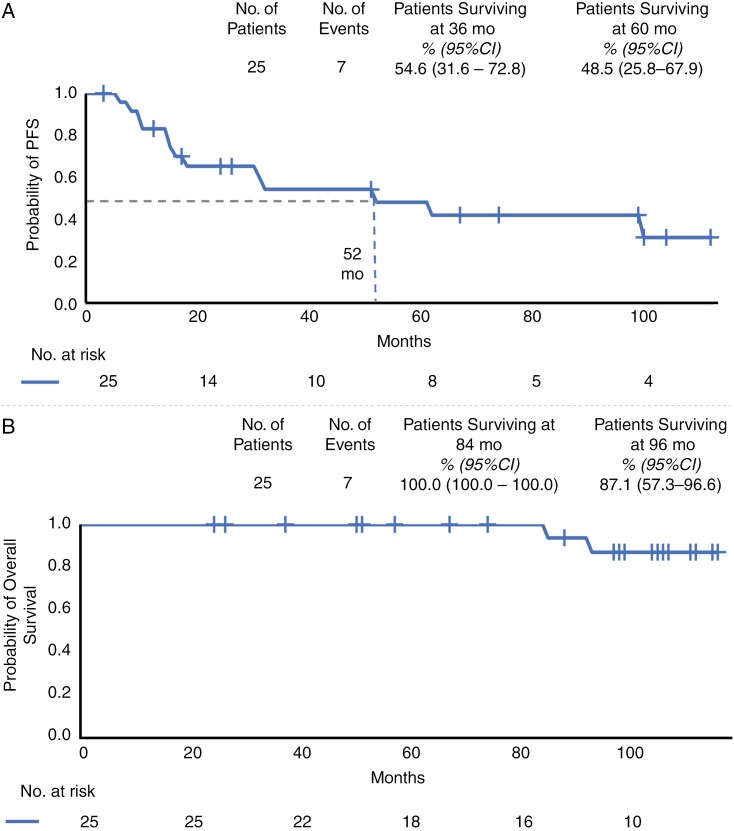
(A) Progression-free survival curve of 25 patients, (B) OS curve of 25 patients.

Of the 25 patients, 10 (40%) had stable disease (SD), and 2 patients (8%) had complete responses. Five patients (20%) were in partial remission (PR), 4 patients (16%) had a minor response (MR), and 4 patients (16%) had progressive disease. Therefore, the response rate (CR + PR + MR) was 44%, and the disease stabilization rate (CR + PR + MR + SD) was 84%. The mean follow-up time was 43 months. A flow diagram of the treatment response is shown in Figure 1. The 3-year PFS rate was 54.6 % (95% CI = 31.6%–72.8%). The 5-year PFS rate was 48.5% (95% CI = 25.8%–67.9%; Figure 2A).

**Figure 3.**
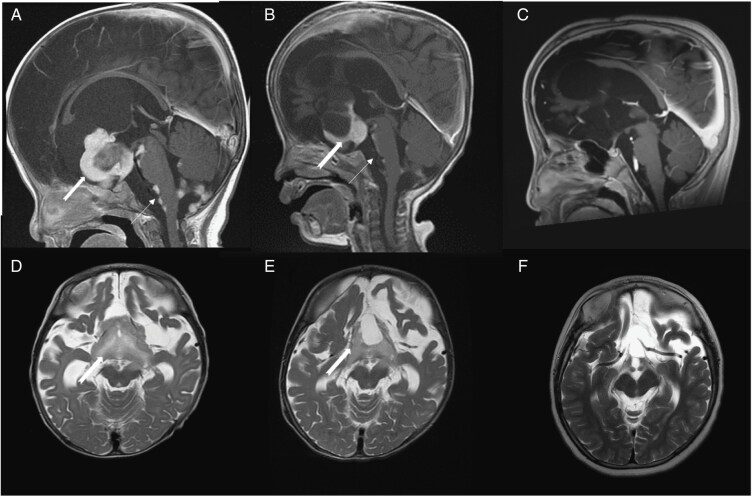
Image of a case that achieved a complete response after receiving carboplatin and vinblastine treatment. (A) T1-weighted postcontrast MR image. Sagittal view of the case prior to chemotherapy in June 2014. The Sella region OPHG is indicated by the thick arrow, and the area of multiple leptomeningeal dissemination is indicated by the thin arrow. (B) A sagittal image was taken 6 months later (after 6 cycles) following chemotherapy. The OPHG (thick arrow) shows a trend of reduction, and the leptomeningeal dissemination (thin arrow) also demonstrates a similar trend of decrease. (C) The most recent sagittal image was taken in September 2023. It shows no signs of recurrence since achieving a complete response. (D, E, F) T2-Flair MR image. Axial view from before treatment (D). six months later (E) and the most recent follow-up in September 2023 (F), which shows no recurrence.

Figure 3 presents a case that achieved CR following chemotherapy. This 6-month-old male baby was diagnosed with OPHG with leptomeningeal dissemination in 2014 (Figure 3A, D) and began monthly treatment with carboplatin and vinblastine in June 2014. After approximately 6 cycles, signs of tumor and leptomeningeal dissemination reduction were observed (Figure 3B, E). The patient subsequently achieved CR and remained relapse-free as of the last follow-up in October 2023 (Figure 3C, F).

The OS results are shown in Figure 2B. The 8-year survival rate was 87.1% (95% CI = 57.3%–96.6%). One patient was initially treated with a different regimen for OPHGs but experienced tumor relapse. Subsequently, the treatment was switched to a monthly carboplatin and vinblastine regimen, with the patient completing a total of 21 cycles and achieving partial regression of the tumor. The tumor relapsed 1 year after completing the carboplatin and vinblastine regimen, and the patient subsequently began a chemotherapy regimen different from the monthly carboplatin and vinblastine protocol. The patient later died 7 years after starting carboplatin and vinblastine treatment. The definite cause of death remains unknown, as the patient was found to have died suddenly at home. It is speculated that the cause of death might have been related to tumor bleeding or other cardiovascular events. The other patient died at 93 months. This patient was diagnosed with myelodysplastic syndrome and died due to severe infection.

**Figure 4.**
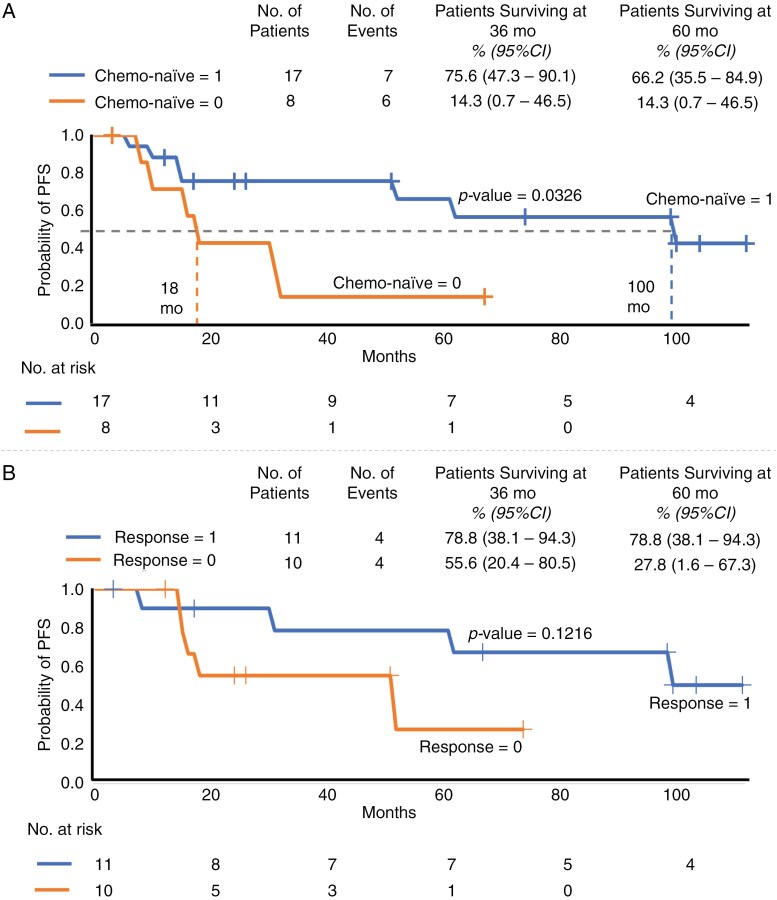
(A) progression-free survival curve between the chemotherapy-naïve group and non-naïve group, (B) progression-free survival curve between response group and stabilization group.

In the univariant analysis, we considered factors such as sex, histology, age at the start of treatment with carboplatin and vinblastine (whether younger or older than 5 years old), tumor resection before chemotherapy, and leptomeningeal dissemination. No significant differences were found between these groups. However, the univariate analysis revealed a higher hazard ratio (3.256, *P *= .0449) in the non-naïve group compared to the chemotherapy-naïve group. The chemotherapy-naïve group showed significantly better PFS than the non-naïve group in the log-rank test (*P *= .0326; Figure 4C). The 3-year PFS rate in the chemotherapy-naïve group was 75.6% (95% CI = 47.3%–90.1%), and the 5-year PFS rate was 66.2% (95% CI = 35.5%–84.9%). We also compared the PFS between the response group (defined as CR, PR, and MR) and the stabilization group (the SD group; Figure 4D), but no significant difference was found between these groups (*P = *.1216).

### Toxicity

The distribution of side effects is provided in Supplementary Table 1. The most prevalent adverse effects included hematopoietic issues, nausea and vomiting, allergy, fever, and infection. Allergic reactions manifested in 10 patients, which were all related to carboplatin and typically occurred between cycles 3 and 21 (median onset: 10 cycles). Severe allergies necessitated cessation of carboplatin combined with vinblastine for 2 patients, who switched to vinblastine monotherapy. Management with antihistamines and corticosteroids effectively mitigated allergic symptoms in the remaining 8 patients, allowing continued chemotherapy. Additionally, one patient prematurely terminated treatment at cycle 18 due to suspected carboplatin-related hearing impairment.

Hematopoietic issues were observed and predominantly involved mild severity (grade < 3). Specifically, 13 patients experienced neutropenia, 18 had anemia, and 13 had thrombocytopenia. Among these, 1 patient had grade 3 neutropenia, which necessitated treatment with antibiotics for neutropenic fever. One patient had grade 3 anemia and received a packed red-blood-cell transfusion accordingly. None of the patients required granulocyte colony-stimulating factor (G-CSF) or platelet transfusion. These findings indicate a manageable spectrum of hematopoietic toxicity associated with carboplatin and vinblastine therapy in this cohort.

Eight patients experienced fever or infection during chemotherapy. Six patients had infections with grade 3 severity. Specifically, one patient developed neutropenic fever and received cefepime. Three patients were diagnosed with port-a-cath infections necessitating surgical removal and intravenous antibiotic therapy. After successful infection control, chemotherapy was resumed.

One patient was diagnosed with a urinary tract infection and treated with intravenous antibiotics during their chemotherapy course. Another patient had bacteremia and received intravenous antibiotics. The remaining 2 patients experienced fever without a definitive infection focus and were managed symptomatically with oral antibiotics.

### Vision

Two patients’ visual acuity could not be assessed sufficiently due to young age and poor cooperation. Two patients’ visual data were lost. In the remaining 21 patients, 6 patients (28.6%) had improved visual acuity, 11 patients (52.4%) had stable visual acuity, and 4 patients (19.0%) had worse visual acuity.

## Discussion

Numerous studies have investigated various chemotherapy regimens for pLGGs, and most series have reported a 5-year PFS rate of around 30%–50%.^16–22^ Among the most commonly utilized chemotherapy protocols, the reported 5-year PFS rates for carboplatin and vincristine are between 39% and 47%,^16–18^ and that of vinblastine monotherapy is between 42.3% and 53.2%.^19,20^ Monthly carboplatin and TPCV have PFS rates of 51% and 52%, respectively.^18,21,22^ A retrospective study investigated a regimen of carboplatin (400 mg/m²) on day 1 combined with vinblastine (4.0 mg/m²) administered weekly for 3 doses every 4 weeks for the treatment of pediatric low-grade glioma.^26^ This regimen achieved a 3-year PFS rate of 39.4% and a 5-year PFS rate of 34.5%, which are comparable to the 5-year PFS rate of 39% observed in previous studies using the carboplatin and vincristine regimen. Studies have consistently demonstrated high OS rates at both 3 and 5 years, which generally exceed 80%.

In comparison, our studies have observed a 5-year PFS rate of 48.5% (95% CI = 25.8–67.9%) and a notably higher 5-year PFS rate of 66.2% (95% CI = 35.5–84.9%) in the chemotherapy-naïve group. In the present study, both the 3-year and 5-year OS rates were 100%. Our dose-reduced, monthly regimen showed similar efficacy, consistent with previous studies, as summarized in Table 2.

**Table 2.** Comparison of Response and PFS Between Different Regimens

Study	Regimen	No.	Response rate%	SD%	Survival	
Our study	Monthly Carboplatin Vinblastine	25(17ND8R)	44	84	3-yr PFS: 54.6% 5-yr PFS: 48.5%	5-yr OS: 100% 8-yr OS: 87.1%
Packer et al, 1997^16^	Weekly Carboplatin Vincristine	78ND	56	94	3-yr PFS: 68%	3-yr OS: 97%
Gnekow et al, 2012^17^	Weekly Carboplatin Vincristine	216 (117ND99R)	35	92	5-yr PFS: 47% 10-yr PFS: 44%	10-yr OS: 88%
Ater et al, 2012^18^	Weekly Carboplatin Vincristine	137ND	50	68	5-yr PFS: 39%	5-yr OS: 86%
Ater et al,2012^18^	TPCV	137ND	56	68	5-yr PFS: 52%	5-yr OS: 87%
Bouffet et al, 2012^19^	Weekly Vinblastine	51R	36	74	5-yr PFS: 42.3%	5-yr OS: 93.2%
Lassaletta et al, 2016^20^	Weekly Vinblastine	54ND	26	87	5-yr PFS: 53.2%	5-yr OS: 94.4%
Dodgshun et al, 2016^21^	Monthly Carboplatin	104ND	10	86	3-yr PFS: 66% 5-yr PFS: 51%	5-yr OS: 97%
Gururangan et al, 2002^22^	Monthly Carboplatin	81(60ND21R)	29	86	3-yr PFS: 64%	3-yr OS: 84%
Nellan et al, 2020^26^	Carboplatin and vinblastine	46 ND	20	74	3-yr PFS: 39.4% 5-yr PFS: 34.5%	5-yr OS: 92%

No., Number of patients; SD%, stabilization rate; PFS, progression survival rate; OS, Overall survival rate.

The response assessment showed a response rate of 44% and a disease stabilization rate of 84%. Previous studies have reported varying response and stabilization rates for different regimens. For instance, carboplatin and vincristine regimens have shown response rates between 35 and 56% and disease stabilization rates between 68 and 94%.^16–18^ Vinblastine monotherapy has shown response rates of 26% and 36% and stabilization rates of 74% and 87%.^19,20^ The TPVC regimen has demonstrated a response rate of 52% and a stabilization rate of 68%, while single-agent carboplatin has shown a response rate of 10% and 29% and a stabilization rate of 86%.^18,21,22^ A previous carboplatin and vinblastine regimen showed a response rate of 20% and a stabilization rate of 74%.^26^Table 2 provides a detailed comparison and indicates that our observed response and stabilization rates for carboplatin and vinblastine are generally non-inferior to those of other treatments.

The non-naïve group showed a higher hazard ratio (3.256, *P = *.0449) compared with the chemotherapy-naïve group. The PFS was significantly higher in the chemotherapy-naïve group than the non-naïve group (*P-*value = .0326). This suggests that being chemotherapy-naïve or non-naïve could serve as a predictive factor of PFS during treatment with carboplatin and vinblastine.

Visual preservation is a critical goal when treating OPHG patients. A systematic review by Moreno et al. identified 174 patients with documented visual outcomes.^31^ The results indicated that following chemotherapy, 25 patients (14.4%) showed improvement in vision, 82 (47.1%) had stabilization, and 67 (38.5%) experienced deterioration. In the present retrospective study, vision data were well documented for 21 patients, and our findings were consistent with previous results, supporting the efficacy of carboplatin and vinblastine in maintaining visual function.

Regimens involving carboplatin have been notably criticized for their allergic reactions and hematotoxicity.^16–18,21,22^ Studies on carboplatin for pLGG have shown significant variability in hypersensitivity rates ranging from 6% to 68%.^32^ A study on vinblastine monotherapy showed that the primary adverse event associated with vinblastine was hematotoxicity, and the most frequent grade 3 and 4 adverse event was neutropenia (22 patients (40.7%) and 19 patients (35.2%), respectively). Only 13 patients (24.1%) tolerated the planned dose of vinblastine (6 mg/m^2^ per week) throughout the entire study.^20^

In our study, patients generally experienced lower rates of hematotoxicity with milder severity compared to those treated with weekly vinblastine monotherapy or a combination of carboplatin and vincristine. Specifically, only one patient (4%) had grade 3 neutropenia, which required antibiotics for neutropenic fever, and another patient (4%) had grade 3 anemia, which necessitated a packed red blood cell transfusion. Allergy to carboplatin remained a significant concern, with 10 out of 25 patients (40%) experiencing allergic reactions. However, 8 of these patients (32%) experienced relief from their allergic symptoms and were able to continue chemotherapy after receiving antihistamine or steroid treatment. Two patients (8%) discontinued carboplatin and switched to weekly vinblastine monotherapy as a result, and one patient (4%) switched due to carboplatin-induced hearing impairment. Despite these challenges, the majority of patients (22 patients, 88%) tolerated the chemotherapy well.

We observed relatively mild hypersensitivity compared to conventional carboplatin and vincristine regimens. However, hypersensitivity caused by carboplatin remains a significant issue. In terms of incidence, the rate of hypersensitivity in our study was not lower than that reported for conventional regimens, as supported by previous research. For example, in a retrospective study comparing weekly and monthly administration of carboplatin, Lafay-Cousin et al. found no significant difference in the incidence of hypersensitivity reactions, though the onset of hypersensitivity occurred earlier with weekly administration.^33^

In our study, most hypersensitivity reactions were relatively low-grade. This observation may be attributed to our use of relatively low-dose carboplatin and the monthly administration schedule. However, given the limitations of a retrospective analysis and the relatively small sample size in our study, we cannot definitively conclude that this regimen offers any advantage over conventional regimens in terms of hypersensitivity.

In addition to the hypersensitivity findings, we also observed significantly milder hematotoxicity compared to weekly vinblastine and the conventional carboplatin and vincristine regimens. Specifically, the monthly and low dosing schedule likely contributed to the lower severity of hematotoxicity. Overall, our findings suggest that monthly carboplatin and vinblastine are generally well tolerated by most patients and show milder or at least non-inferior toxicity profiles compared to previous treatments involving carboplatin and vincristine and vinblastine monotherapy.

Our findings also compared favorably with those of a phase 2 trial of selumetinib (a MEK1/2 inhibitor), which involved 25 children with recurrent OPHG without NF1. The trial reported a response rate of 24% (only complete or partial responses were considered as responses, and MRs were excluded). The 2-year PFS was 73.8%. In this study, 21% of patients had improved visual acuity, and 68% had stable acuity. The most common toxicities were elevated creatine phosphokinase (CPK), anemia, diarrhea, headache, nausea, emesis, fatigue, and elevated aspartate transaminase (AST)/alanine transaminase (ALT). Rare adverse events of grade 3 or 4 were noted.^9^ That study focused exclusively on recurrent cases without long-term survival data (3- and 5-year PFS). Our study showed comparable response rates and visual preservation, which further underscore the efficacy of our regimen.

There are some limitations to our study. First, this study was retrospective and probably impacted by selection bias. Most similar studies had a much higher number of patients and included cases of pLGGs that were located in other regions in addition to the optic pathway and hypothalamus region. However, only OPHG patients were included in our study, and the total number was only 25. This could explain why the 95% CI regarding the 3- and 5-year of PFS was wide. It could cause the estimation to become less precise.

Another limitation is the lack of molecular marker data. As a result, the effect of molecular markers such as BRAF was unknown. Multiple retrospective studies have indicated that patients with tumors carrying the BRAF V600E mutation tend to respond less effectively to chemotherapy and are associated with shorter PFS and OS.^14,34^ This limitation may have impacted the accuracy of our assessment of efficacy, and further research may be needed to explore this issue in more depth. Most patients in this study had Dodge stage 3 OPGHs (88%), only 1 patient had stage 1, and 2 patients had stage 2. Therefore, the correlation between tumor location and PFS could not be determined sufficiently.

## Conclusions

This retrospective study has shown that a regimen of carboplatin and vinblastine monthly is effective for OPHGs and has milder toxicities compared to conventional chemotherapy regimens. Most of the patients can go to school or live a normal life. Therefore, lengthening the interval between treatments is more convenient for both patients and parents. In conclusion, our monthly protocol may be appropriate for patients who need chemotherapy and are not suitable for targeted therapy as a choice of the first line of treatment.

## Supplementary Material

vdaf020_suppl_Supplementary_Table

## Data Availability

We are unable to publicly share the raw data used in this study due to ethical and privacy considerations, as mandated by the Institutional Review Board (IRB) of Taipei Veterans General Hospital. A de-identified and aggregated summary of the data that supports the findings presented in this research can be made available upon reasonable request to the corresponding author, subject to approval by the Research Ethics Review Committee.

## References

[1] Thomas RP , Gibbs IC, Xu LW, Recht L. Treatment options for optic pathway gliomas. Curr Treat Options Neurol. 2015;17(2):333. 25619537 10.1007/s11940-014-0333-2

[2] Rodriguez FJ , Perry A, Gutmann DH, et al Gliomas in neurofibromatosis type 1: A clinicopathologic study of 100 patients. J Neuropathol Exp Neurol. 2008;67(3):240–249. 18344915 10.1097/NEN.0b013e318165eb75 PMC3417064

[3] Listernick R , Charrow J, Greenwald M, Mets M. Natural history of optic pathway tumors in children with neurofibromatosis type 1: A longitudinal study. J Pediatr. 1994;125(1):63–66. 8021787 10.1016/s0022-3476(94)70122-9

[4] Helfferich J , Nijmeijer R, Brouwer OF, et al Neurofibromatosis type 1 associated low grade gliomas: A comparison with sporadic low grade gliomas. Crit Rev Oncol Hematol. 2016;104:30–41. 27263935 10.1016/j.critrevonc.2016.05.008

[5] Goodden J , Pizer B, Pettorini B, et al The role of surgery in optic pathway/hypothalamic gliomas in children. J Neurosurg Pediatr. 2014;13(1):1–12. 24138145 10.3171/2013.8.PEDS12546

[6] Erkal HS , Serin M, Cakmak A. Management of optic pathway and chiasmatic-hypothalamic gliomas in children with radiation therapy. Radiother Oncol. 1997;45(1):11–15. 9364626 10.1016/s0167-8140(97)00102-3

[7] Bandopadhayay P , Bergthold G, London WB, et al Long-term outcome of 4,040 children diagnosed with pediatric low-grade gliomas: An analysis of the Surveillance Epidemiology and End Results (SEER) database. Pediatr Blood Cancer. 2014;61(7):1173–1179. 24482038 10.1002/pbc.24958 PMC4657506

[8] Krishnatry R , Zhukova N, Guerreiro Stucklin AS, et al Clinical and treatment factors determining long-term outcomes for adult survivors of childhood low-grade glioma: A population-based study. Cancer. 2016;122(8):1261–1269. 26970559 10.1002/cncr.29907

[9] Fangusaro J , Onar-Thomas A, Poussaint TY, et al A phase II trial of selumetinib in children with recurrent optic pathway and hypothalamic low-grade glioma without NF1: A Pediatric Brain Tumor Consortium study. Neuro Oncol. 2021;23(10):1777–1788. 33631016 10.1093/neuonc/noab047 PMC8485450

[10] Fangusaro J , Onar-Thomas A, Young Poussaint T, et al Selumetinib in paediatric patients with BRAF-aberrant or neurofibromatosis type 1-associated recurrent, refractory, or progressive low-grade glioma: A multicentre, phase 2 trial. Lancet Oncol. 2019;20(7):1011–1022. 31151904 10.1016/S1470-2045(19)30277-3 PMC6628202

[11] Karajannis MA , Legault G, Fisher MJ, et al Phase II study of sorafenib in children with recurrent or progressive low-grade astrocytomas. Neuro Oncol. 2014;16(10):1408–1416. 24803676 10.1093/neuonc/nou059 PMC4165419

[12] Banerjee A , Jakacki RI, Onar-Thomas A, et al A phase I trial of the MEK inhibitor selumetinib (AZD6244) in pediatric patients with recurrent or refractory low-grade glioma: A Pediatric Brain Tumor Consortium (PBTC) study. Neuro Oncol. 2017;19(8):1135–1144. 28339824 10.1093/neuonc/now282 PMC5570236

[13] Bouffet E , Hansford JR, Garrè ML, et al Dabrafenib plus trametinib in pediatric glioma with BRAF V600 mutations. N Engl J Med. 2023;389(12):1108–1120. 37733309 10.1056/NEJMoa2303815

[14] Ryall S , Zapotocky M, Fukuoka K, et al Integrated molecular and clinical analysis of 1,000 pediatric low-grade gliomas. Cancer Cell. 2020;37(4):569–583.e5. 32289278 10.1016/j.ccell.2020.03.011 PMC7169997

[15] Schindler G , Capper D, Meyer J, et al Analysis of BRAF V600E mutation in 1,320 nervous system tumors reveals high mutation frequencies in pleomorphic xanthoastrocytoma, ganglioglioma and extra-cerebellar pilocytic astrocytoma. Acta Neuropathol. 2011;121(3):397–405. 21274720 10.1007/s00401-011-0802-6

[16] Packer RJ , Ater J, Allen J, et al Carboplatin and vincristine chemotherapy for children with newly diagnosed progressive low-grade gliomas. J Neurosurg. 1997;86(5):747–754. 9126887 10.3171/jns.1997.86.5.0747

[17] Gnekow AK , Falkenstein F, von Hornstein S, et al Long-term follow-up of the multicenter, multidisciplinary treatment study HIT-LGG-1996 for low-grade glioma in children and adolescents of the German Speaking Society of Pediatric Oncology and Hematology. Neuro Oncol. 2012;14(10):1265–1284. 22942186 10.1093/neuonc/nos202 PMC3452343

[18] Ater JL , Zhou T, Holmes E, et al Randomized study of two chemotherapy regimens for treatment of low-grade glioma in young children: A report from the Children’s Oncology Group. J Clin Oncol. 2012;30(21):2641–2647. 22665535 10.1200/JCO.2011.36.6054 PMC3413276

[19] Bouffet E , Jakacki R, Goldman S, et al Phase II study of weekly vinblastine in recurrent or refractory pediatric low-grade glioma. . J Clin Oncol. 2012;30(12):1358–1363. 22393086 10.1200/JCO.2011.34.5843

[20] Lassaletta A , Scheinemann K, Zelcer SM, et al Phase II weekly vinblastine for chemotherapy-naïve children with progressive low-grade glioma: A Canadian Pediatric Brain Tumor Consortium study. J Clin Oncol. 2016;34(29):3537–3543. 27573663 10.1200/JCO.2016.68.1585

[21] Dodgshun AJ , Maixner WJ, Heath JA, Sullivan MJ, Hansford JR. Single agent carboplatin for pediatric low-grade glioma: A retrospective analysis shows equivalent efficacy to multiagent chemotherapy. Int J Cancer. 2016;138(2):481–488. 26235348 10.1002/ijc.29711

[22] Gururangan S , Cavazos CM, Ashley D, et al Phase II study of carboplatin in children with progressive low-grade gliomas. J Clin Oncol. 2002;20(13):2951–2958. 12089224 10.1200/JCO.2002.12.008

[23] Rosca L , Robert-Boire V, Delisle JF, Samson Y, Perreault S. Carboplatin and vincristine neurotoxicity in the treatment of pediatric low-grade gliomas. Pediatr Blood Cancer. 2018;65(11):e27351. 30014595 10.1002/pbc.27351

[24] Nelson RL. The comparative clinical pharmacology and pharmacokinetics of vindesine, vincristine, and vinblastine in human patients with cancer. Med Pediatr Oncol. 1982;10(2):115–127. 7070351 10.1002/mpo.2950100202

[25] Jakacki RI , Bouffet E, Adamson PC, et al A phase 1 study of vinblastine in combination with carboplatin for children with low-grade gliomas: A Children’s Oncology Group phase 1 consortium study. Neuro Oncol. 2011;13(8):910–915. 21764821 10.1093/neuonc/nor090 PMC3145477

[26] Nellan A , Wright E, Campbell K, et al Retrospective analysis of combination carboplatin and vinblastine for pediatric low-grade glioma. J Neurooncol. 2020;148(3):569–575. 32506370 10.1007/s11060-020-03549-x

[27] Dodge HW, Jr, Love JG, Craig WM, et al Gliomas of the optic nerves. AMA Arch Neurol Psychiatry. 1958;79(6):607–621. 13532071 10.1001/archneurpsyc.1958.02340060003001

[28] Wen PY , van den Bent M, Youssef G, et al RANO 2.0: Update to the response assessment in neuro-oncology criteria for high- and low-grade gliomas in adults. J Clin Oncol. 2023;41(33):5187–5199. 37774317 10.1200/JCO.23.01059 PMC10860967

[29] Taylor T , Jaspan T, Milano G, et al; PLAN Study Group. Radiological classification of optic pathway gliomas: Experience of a modified functional classification system. Br J Radiol. 2008;81(970):761–766. 18796556 10.1259/bjr/65246351

[30] Walker DA , Liu J, Kieran M, et al; CPN Paris 2011 Conference Consensus Group. A multi-disciplinary consensus statement concerning surgical approaches to low-grade, high-grade astrocytomas and diffuse intrinsic pontine gliomas in childhood (CPN Paris 2011) using the Delphi method. Neuro Oncol. 2013;15(4):462–468. 23502427 10.1093/neuonc/nos330 PMC3607269

[31] Moreno L , Bautista F, Ashley S, Duncan C, Zacharoulis S. Does chemotherapy affect the visual outcome in children with optic pathway glioma? A systematic review of the evidence. Eur J Cancer. 2010;46(12):2253–2259. 20400294 10.1016/j.ejca.2010.03.028

[32] Dodgshun AJ , Hansford JR, Cole T, Choo S, Sullivan MJ. Carboplatin hypersensitivity reactions in pediatric low grade glioma are protocol specific and desensitization shows poor efficacy. Pediatr Blood Cancer. 2016;63(1):17–20. 26207610 10.1002/pbc.25686

[33] Lafay-Cousin L , Sung L, Carret AS, et al Carboplatin hypersensitivity reaction in pediatric patients with low-grade glioma: A Canadian Pediatric Brain Tumor Consortium experience. Cancer. 2008;112(4):892–899. 18098210 10.1002/cncr.23249

[34] Lassaletta A , Zapotocky M, Mistry M, et al Therapeutic and prognostic implications of BRAF V600E in pediatric low-grade gliomas. J Clin Oncol. 2017;35(25):2934–2941. 28727518 10.1200/JCO.2016.71.8726 PMC5791837

