# Supplementary material for: Carboplatin and vinblastine monthly in the optic pathway and hypothalamic gliomas: A retrospective analysis in a single institute
Source: Neurooncol Adv. 2025 Jan 29;7(1):vdaf020. doi: 10.1093/noajnl/vdaf020 (PMC11883347; doi:10.1093/noajnl/vdaf020)
Supplement: vdaf020_suppl_Supplementary_Table [file vdaf020_suppl_supplementary_table.docx]

Table 1 Toxicity distribution

| Adverse event | No. of patients  Total | No. of patients  $\boldsymbol{\geq}$ grade3 |
| --- | --- | --- |
| Hematopoietic |  |  |
| Neutropenia | 13 (52%) | 1 (4%) |
| Anemia | 18 (72%) | 1 (4%) |
| Thrombocytopenia | 13 (52%) | 0 |
| Nausea and vomiting | 14 (56%) | 0 |
| Hypersensitivity | 10 (40%) | 2 (8%) |
| Fever and infection | 8 (32%) | 6 (24%) |
| Anorexia | 7 (28%) | 0 |
| Peripheral sensory disturbance | 5 (20%) | 0 |
| Increased AST/ALT | 3 (12%) | 0 |
| Mucositis | 2 (8%) | 0 |
| Hyperkalemia | 2 (8%) | 0 |
| Hypernatremia | 1 (4%) | 0 |
| Hearing impairment | 1 (4%) | 1(4%) |
